# Assessment of main adverse drug reactions in systematic reviews and clinical trials of heparins for surgery prophylaxis

**DOI:** 10.1186/1745-6215-16-S3-P1

**Published:** 2015-11-24

**Authors:** Daniela R Junqueira

**Affiliations:** 1 School of Physiotherapy, Universidade Federal de Minas Gerais (UFMG), Belo Horizonte, Brazil

## Background

In September 2012, we published a Cochrane systematic review assessing the risk of heparin-induced thrombocytopenia (HIT) in postoperative patients. Together with haemorrhagic events, HIT is a main adverse reaction of heparins and its most important consequence is a paradoxical increase in the risk of thromboembolic complications.

## Method

We evaluated the report of HIT in Cochrane reviews of unfractionated heparin (UFH) or low molecular weight heparins (LMWH) for thromboprophylaxis after any type of surgery from the 2013 to 2015, period after the publication of a Cochrane review focused on the frequency of HIT in surgical settings. Data extraction aimed to describe how often and accurately HIT was addressed as a specific outcome (primary or secondary) with a precise definition, and how complete was the report of the included clinical trials regarding HIT.

## Results

Four reviews were identified, each one relating to different clinical settings: cancer patients undergoing surgery (n=1), retinal reattachment surgery (n=1), microvascular surgery for digital replantation (n=1), and major amputation of lower extremity (n=1). Only one review described HIT as secondary outcome and none of the reviews indicated the accepted definition of HIT when outlining the outcomes of interest. A total of 22 clinical trials were included in the reviews, comprising a total of 14,120 patients, but no report of HIT was described.

## Conclusion

Systematic reviewers need to be aware of special concepts and definitions in order to collate quality and accurate data not just related to the efficacy of the drug interventions but also in relation of its safety. Considering the relevance, bleeding and HIT should be regarded as core outcomes for the assessment of the safety of heparins. However, the complex definition and testing requirements for the diagnosis of HIT may determine significant bias in the detection and reporting of this adverse drug reaction.

